# A Benchmark of Data Stream Classification for Human Activity Recognition on Connected Objects

**DOI:** 10.3390/s20226486

**Published:** 2020-11-13

**Authors:** Martin Khannouz, Tristan Glatard

**Affiliations:** Department of Computer Science and Software Engineering, Concordia University, Montréal, QC H3G 1M8, Canada; tristan.glatard@concordia.ca

**Keywords:** application platform, data management and analytics, smart environment, data streams, classification, power, memory footprint, benchmark, human activity recognition, MCNN, Mondrian, Hoeffding tree

## Abstract

This paper evaluates data stream classifiers from the perspective of connected devices, focusing on the use case of Human Activity Recognition. We measure both the classification performance and resource consumption (runtime, memory, and power) of five usual stream classification algorithms, implemented in a consistent library, and applied to two real human activity datasets and three synthetic datasets. Regarding classification performance, the results show the overall superiority of the Hoeffding Tree, the Mondrian forest, and the Naïve Bayes classifiers over the Feedforward Neural Network and the Micro Cluster Nearest Neighbor classifiers on four datasets out of six, including the real ones. In addition, the Hoeffding Tree and—to some extent—the Micro Cluster Nearest Neighbor, are the only classifiers that can recover from a concept drift. Overall, the three leading classifiers still perform substantially worse than an offline classifier on the real datasets. Regarding resource consumption, the Hoeffding Tree and the Mondrian forest are the most memory intensive and have the longest runtime; however, no difference in power consumption is found between classifiers. We conclude that stream learning for Human Activity Recognition on connected objects is challenged by two factors which could lead to interesting future work: a high memory consumption and low F1 scores overall.

## 1. Introduction

Internet of Things applications may adopt a centralized model, where connected objects transfer data to servers with adequate computing capabilities, or a decentralized model, where data are analyzed directly on the connected objects or on nearby devices. While the decentralized model limits network transmission, increases battery life [1,2], and reduces data privacy risks, it also raises important processing challenges due to the modest computing capacity of connected objects. Indeed, it is not uncommon for wearable devices and other smart objects to include a processing memory of less than 100 KB, little to no storage memory, a slow CPU, and no operating system. With multiple sensors producing data at a high frequency, typically 50 Hz to 800 Hz, processing speed and memory consumption become critical properties of data analyses.

Data stream processing algorithms are precisely designed to analyze virtually infinite sequences of data elements with reduced amounts of working memory. Several classes of stream processing algorithms were developed in past decades, such as filtering, counting, or sampling algorithms [3]. These algorithms must follow multiple constraints such as a constant processing time per data element, or a constant space complexity [4]. Our study focuses on supervised classification, a key component of contemporary data models.

We evaluate supervised data stream classifiers from the point of view of connected objects, with a particular focus on Human Activity Recognition (HAR). The main motivating use case is that of wearable sensors measuring 3D acceleration and orientation at different locations on the human body, from which activities such as gym exercises have to be predicted. A previously untrained supervised classifier is deployed directly on the wearables or on a nearby object, perhaps a watch, and aggregates the data, learns a data model, predicts the current activity, and episodically receives true labels from the human subject. Our main question is to determine whether on-chip classification is feasible in this context.

We evaluate existing classifiers from the complementary angles of (1) classification performance, including in the presence of concept drift, and (2) resource consumption, including memory usage and classification time per element (latency). We consider six datasets in our benchmark, including three that are derived from the two most popular open datasets used for HAR, and three simulated datasets.

Compared to the previous works reviewed in Section 2, the contributions of our paper are the following:

- We compare the most popular data stream classifiers on the specific case of HAR;
- We provide quantitative measurements of memory and power consumption, as well as runtime;
- We implement data stream classifiers in a consistent software library meant for deployment on embedded systems.

The subsequent sections present the Materials, Methods, and Results of our benchmark.

## 2. Related Work

To the best of our knowledge, no previous study focused on the comparison of data stream classifiers for HAR in the context of limited memory and available runtime that characterizes connected objects.

### 2.1. Comparisons of Data Stream Classifiers

Data stream classifiers were compared mostly using synthetic datasets or real but general-purpose ones (Electrical, CoverType, Poker), which is not representative of our use case. In addition, memory and runtime usage are rarely reported, with the notable exception of [5].

The work in [6] reviews an extensive list of classifiers for data streams, comparing the Hoeffding Tree, the Naïve Bayes, and the k-nearest neighbor (*k*-NN) online classifiers. The paper reports an accuracy of 92 for online *k*-NN, 80 for the Hoeffding Tree, and 60 for Naïve Bayes. The study is limited to a single dataset (CoverType).

The work in [7] compares four classifiers (Bayesnet, Hoeffding Tree, Naïve Bayes, and Decision Stump) using synthetic datasets. It reports a similar accuracy of 90 for the Bayesnet, the Hoeffding Tree, and Naïve Bayes classifiers, while the Decision Stump one only reaches 65. Regarding runtimes, Bayesnet is found to be four times slower than the Hoeffding Tree which is itself three times slower than Naïve Bayes and Decision Stump.

The work in [8] compares ensemble classifiers on imbalanced data streams with concept drifts, using two real datasets (Electrical, Intrusion), synthetic datasets, and six classifiers, including the Naïve Bayes and the Hoeffding Tree ones. The Hoeffding Tree is found to be the second most accurate classifier after the Accuracy Updated Ensemble.

The authors in [9] have analyzed the resource trade-offs of six online decision trees applied to edge computing. Their results showed that the Very Fast Decision Tree and the Strict Very Fast Decision Tree were the most energy friendly, the latter having the smallest memory footprint. On the other hand, the best predictive performances were obtained in combination with OLBoost. In particular, the paper reports an accuracy of 89.6% on the Electrical dataset, and 83.2% on the Hyperplane dataset.

Finally, the work in [5] describes the architecture of StreamDM-C++ and presents an extensive benchmark of tree-based classifiers, covering runtime, memory, and accuracy. Compared to other tree-based classifiers, the Hoeffding Tree classifier is found to have the smallest memory footprint while the Hoeffding Adaptive Tree classifier is found to be the most accurate on most of the datasets.

### 2.2. Offline and Data Stream Classifiers for HAR

In this study, we focus on HAR conducted from wearable sensors used, for instance, during sport activities. Other works focus on daily human activities such as cooking or cleaning [10,11,12], mostly using home sensors. These studies describe how they have collected datasets from heterogeneous sensor networks located in apartments. Except for the Opportunity datasets shown in [12], the data were collected from sensors placed in various daily objects such as entrance doors or cupboards. The Opportunity dataset has, in addition, wearable sensors placed on the subjects. These papers also provide a baseline F1 score with popular classifiers (Naïve Bayes, *k*-NN, and neural network).

Several other studies evaluated classifiers for HAR with sport activities in an offline (non data stream) setting. In particular, the work in [13] compared 293 classifiers using various sensor placements and window sizes, concluding on the superiority of k nearest neighbors (*k*-NN) and pointing out a trade-off between runtime and classification performance. Resource consumption, including memory and runtime, was also studied for offline classifiers, such as in [14] for the particular case of the R programming language.

In addition, the work in [15] achieved an offline accuracy of 99.4% on a five-class dataset of HAR. The authors used AdaBoost, an ensemble method, with ten offline decision trees. The work in [16] proposes a Support Vector Machine enhanced with feature selection. Using smartphone data, the model showed above 90% accuracy on day-to-day human activities. Finally, the work in [17] applies three offline classifiers to smartphone and smartwatch human activity data. The results show that Convolutional Neural Network and Random Forest achieve F1 scores of 0.98 with smartwatches and 0.99 with smartphones.

In a data stream (online) setting, the work in [18] presents a wearable system capable of running pre-trained classifiers on the chip with high classification accuracy. This shows the superiority of the proposed Feedforward Neural Network over *k*-NN.

In this study, we focus on HAR with wearable sensor data processed with data streams classifiers and we use OrpailleCC, an OS-independent library that could be deployed on connected objects. To the best of our knowledge, this is the first benchmark conducted in this context.

## 3. Materials and Methods

We evaluate 5 classifiers implemented in either StreamDM-C++ [5] or OrpailleCC [19]. StreamDM-C++ is a C++ implementation of StreamDM [20], a software to mine big data streams using https://spark.apache.org/streaming/Apache Spark Streaming. StreamDM-C++ is usually faster than StreamDM in single-core environments, due to the overhead induced by Spark.

OrpailleCC is a collection of data stream algorithms developed for embedded devices. The key functions, such as random number generation or memory allocation, are parametrizable through class templates and can thus be customized on a given execution platform. OrpailleCC is not limited to classification algorithms, it implements other data stream algorithms such as the Cuckoo filter [21] or a multi-dimensional extension of the Lightweight Temporal Compression [22]. We extended it with a few classifiers for the purpose of this benchmark.

This benchmark includes five popular classification algorithms. The Mondrian forest [23] builds decision trees without the immediate need for labels, which is useful in situations where labels are delayed [24]. The Micro-Cluster Nearest Neighbor [25] classifier is a compressed version of the k-nearest neighbor (*k*-NN) classifier that was shown to be among the most accurate classifiers for HAR from wearable sensors [13]. The Naïve Bayes [26] classifier builds a table of attribute occurrence to estimate class likelihoods. The Hoeffding Tree [27] builds a decision tree using the Hoeffding Bound to estimate when the best split is found. Finally, Neural Network classifiers have become popular by reaching or even exceeding human performance in many fields such as image recognition or game playing. We use a Feedforward Neural Network with one hidden layer, as described in [18] for the recognition of fitness activities on a low-power platform.

The remainder of this section details the datasets, classifiers, evaluation metrics and parameters used in our benchmark.

### 3.1. Datasets

To conduct our benchmark, we selected the main two datasets commonly used to evaluate HAR from wearable sensors. In addition, we used the popular Massive Online Analysis (MOA) stream simulator to generate three synthetic datasets with different properties.

#### 3.1.1. Banos et al.

The Banos et al. dataset [28] is a human activity dataset with 17 participants and 9 sensors per participantv (Banos et al.  dataset available https://archive.ics.uci.edu/ml/datasets/REALDISP+Activity+Recognition+Dataset#:~:text=The%20REALDISP%20(REAListic%20sensor%20DISPlacement,%2Dplacement%20and%20induced%2Ddisplacement). Each sensor samples a 3D acceleration, gyroscope, and magnetic field, as well as the orientation in a quaternion format, producing a total of 13 values. Sensors are sampled at 50 Hz, and each sample is associated with one of 33 activities. In addition to the 33 activities, an extra activity labeled 0 indicates no specific activity.

We pre-process the Banos et al. dataset using non-overlapping windows of one second (50 samples), and using only the 6 axes (acceleration and gyroscope) of the right forearm sensor. We compute the average and the standard deviation over the window as features for each axis. We assign the most frequent label to the window. The resulting data points were shuffled uniformly.

In addition, we construct another dataset from Banos et al., in which we simulate a concept drift by shifting the activity labels in the second half of the data stream. This is useful to observe any behavioral change induced by the concept drift such as an increase in power consumption.

#### 3.1.2. Recofit

The Recofit dataset [29] is a human activity dataset containing 94 participants (Recofit dataset available https://msropendata.com/datasets/799c1167-2c8f-44c4-929c-227bf04e2b9a). Similar to the Banos et al. dataset, the activity labeled 0 indicates no specific activity. Since many of these activities were similar, we merged some of them together based on the table in [30].

We pre-processed the dataset similarly to the Banos et al. one, using non-overlapping windows of one second, and only using 6 axes (acceleration and gyroscope) from one sensor. From these 6 axes, we used the average and the standard deviation over the window as features. We assigned the most frequent label to the window.

#### 3.1.3. MOA Datasets

Massive Online Analysis [31] (MOA) is a Java framework to compare data stream classifiers. In addition to classification algorithms, MOA provides many tools to read and generate datasets. We generate three synthetic datasets (MOA commands available https://github.com/big-data-lab-team/benchmark-har-data-stream/blob/f314bf4a258e96e418e249228897d269c59cd522/Makefile#L104): a hyperplane, a RandomRBF, and a RandomTree dataset. We generate 200,000 data points for each of these synthetic datasets. The hyperplane and the RandomRBF both have three features and two classes; however, the RandomRBF has a slight imbalance toward one class. The RandomTree dataset is the hardest of the three, with six attributes and ten classes. Since the data points are generated with a tree structure, we expect the decision trees to show better performances than the other classifiers.

### 3.2. Algorithms and Implementation

In this section, we describe the algorithms used in the benchmark, their hyperparameters, and relevant implementation details. We selected two of the main algorithms in the popular StreamDM library: Naïve Bayes and Hoeffding Tree. These algorithms are commonly found in data stream classification studies. In addition, we selected representative algorithms from the main classfification approaches: the Mondrian forest for tree based-learning, Micro Cluster Nearest Neighbor for cluster based-learning, and a Feedforward Neural Network for neural networks.

#### 3.2.1. Mondrian Forest

Each tree in a Mondrian forest [23] recursively splits the feature space, similar to a regular decision tree. However, the feature used in the split and the value of the split are picked randomly. The probability to select a feature is proportional to its normalized range, and the value for the split is uniformly selected in the range of the feature. During prediction, a node combines its observed label count with its parent prediction. Since the Mondrian tree is able to reshape the internal structure of the tree, we expect the Mondrian forest to recover from concept drifts, although most likely slower than Micro-Cluster Nearest Neighbor (MCNN).

In OrpailleCC, the amount of memory allocated to the forest is predefined, and it is shared by all the trees in the forest, leading to a constant memory footprint for the classifier. This implementation is memory-bounded, meaning that the classifier can adjust to memory limitations, for instance, by stopping tree growth or replacing existing nodes with new ones. This is different from an implementation with a constant space complexity, where the classifier would use the same amount of memory regardless of the amount of available memory. For instance, in our study, the Mondrian forest classifier is memory-bounded while the Naïve Bayes classifier has a constant space complexity.

Mondrian trees can be tuned using three parameters: the base count, the discount factor, and the budget. The base count is used to initialize the prediction for the root. The discount factor influences the nodes on how much they should use their parent prediction. A discount factor closer to one makes the prediction of a node closer to the prediction of its parent. Finally, the budget controls the tree depth.

The hyperparameters used for Mondrian forest are shown in Table 1. Additionally, the Mondrian forest is allocated 600 KB of memory unless specified otherwise. On the Banos et al. and Recofit datasets, we also explore a Mondrian forest with 3 MB of memory in order to observe the effect of available memory on performance (classification, runtime, and power).

#### 3.2.2. Micro-Cluster Nearest Neighbor

The Micro-Cluster Nearest Neighbor (MCNN) [25] is a variant of k-nearest neighbors where data points are aggregated into clusters to reduce storage requirements. During training, the algorithm merges a new data point to the closest cluster that shares the same label. If the closest cluster does not share the same label as the data point, this closest cluster and the closest cluster with the same label are assigned an error. When a cluster receives too many errors, it is split. During classification, MCNN  returns the label of the closest cluster. Regularly, the algorithm also assigns a participation score to each cluster and when this score gets below a threshold, the cluster is removed. Given that the maximum number of clusters is fixed, this mechanism makes space for new clusters, and possibly helps adjust to concept drifts. The space and time complexities of MCNN are constant since the maximum number of clusters is fixed. The reaction to concept drift is influenced by the participation threshold and the error threshold. A higher participation threshold and a lower error threshold increase the reaction speed to concept drift. Since the error thresholds used in this study are small, we expect MCNN to react quite quickly and efficiently to concept drifts.

We implemented two versions of MCNN in OrpailleCC, differing in the way they remove clusters during training. The first version (MCNN Origin) is similar to the mechanism described in [25], based on participation scores. The second version (MCNN OrpailleCC) removes the cluster with the lowest participation only when space is needed. A cluster slot is needed when an existing cluster is split and there are no more slots available because the number of active clusters already reached the maximum defined by the user.

MCNN OrpailleCC has only one parameter: the error threshold after which a cluster is split. MCNN Origin has two parameters: the error threshold and the participation threshold. The participation threshold is the limit below which a cluster is removed. The hyperparameters used for MCNN are shown in Table 2. Additionally, both implementations have the cluster count as their last parameter.

#### 3.2.3. Naïve Bayes

The Naïve Bayes [26] algorithm keeps a table of counters for each feature value and each label. During prediction, the algorithm assigns a score for each label depending on how the data point to predict compares to the values observed during the training phase. Since the counters are not biased toward the more recent data points, we expect Naïve Bayes to be slow to adapt if not ineffective in a concept drift situation.

The implementation from StreamDM-C++ was used in this benchmark. It uses a Gaussian fit for numerical attributes. Two implementations were used, the OrpailleCC one and the StreamDM one. We used two implementations to provide a comparison reference between the two libraries.

#### 3.2.4. Hoeffding Tree

Similar to a decision tree, the Hoeffding Tree [27] recursively splits the feature space to maximize a metric, often the information gain or the Gini index. However, to estimate when a leaf should be split, the Hoeffding Tree relies on the Hoeffding bound, a measure of the score deviation of the splits. This measure allows the leaf to decide when the best split is clearly better than the second best split based on the data observed from the stream. This mechanism prevents the tree from waiting until the end of the stream to ensure a split is the best. During classification, a data point is sorted to a leaf, and a label is predicted by aggregating the labels of the training data points in that leaf, usually through majority voting or Naïve Bayes classification. We used this classifier as implemented in StreamDM-C++. The Hoeffding Tree is common in data stream classification; however, the internal nodes are static and cannot be re-considered. Therefore, any concept drift adaption relies on the new leaves that will be split.

The Hoeffding Tree has three parameters: the confidence level, the grace period, and the leaf learner. The confidence level is the probability that the Hoeffding bound makes a wrong estimation of the deviation. The grace period is the number of processed data points before a leaf is evaluated for a split. The leaf learner is the method used in the leaf to predict the label. In this study, we used a confidence level of 0.01 with a grace period of 10 data points and the Naïve Bayes classifier as a leaf learner.

#### 3.2.5. Feedforward Neural Network

A neural network is a combination of artificial neurons, also known as perceptrons, that all have input weights and an activation function. To predict a class label, the perceptron applies the activation function to the weighted sum of its input values. The output value of the perceptron is the result of this activation function. This prediction phase is also called feedforward. To train the neural network, feedforward is applied first, then the error between the prediction and the expected result is used in the backpropagation process to adjust the weights of the input values. A neural network combines multiple perceptrons by connecting perceptron outputs to inputs of other perceptrons. In this benchmark, we used a fully connected Feedforward Neural Network, that is, a network where perceptrons are organized in layers and all output values from perceptrons of layer n−1 serve as input values for perceptrons of layer *n*. We used a 3-layer network with 120 inputs, 30 perceptrons in the hidden layer, and 33 output perceptrons. Because a Feedforward Neural Network takes many epochs to update and converge, it barely adapts to concept drifts even though it trains with each new data point.

In this study, we used histogram features from [18] instead of the ones presented in Section 3.1 because the network performed poorly with these features. The histogram features produce 20 bins per axis.

This neural network can be tuned by changing the number of layers and the size of each layer. Additionally, the activation function and the learning ratio can be changed. The learning ratio indicates by how much the weights should change during backpropagation.

#### 3.2.6. Hyperparameter Tuning

For each classifier, we tuned the hyperparameters using the first subject from the Banos et al. dataset. The data from this subject were pre-processed as the rest of the Banos et al. dataset (window size of one second, average and standard deviation on the six-axis of the right forearm sensor, *…*). We did a grid search to test multiple values for the parameters.

The classifiers start the prequential phase with no knowledge from the first subject. We made an exception for the Feedforward Neural Network because we noticed that it performed poorly with random weights and it needed many epochs to achieve better performances than a random classifier. Therefore, we decided to pre-train the Feedforward Neural Network and re-use the weights as a starting point for the prequential phase.

For other classifiers, only the hyperparameters were taken from the tuning phase. We selected the hyperparameters that maximized the F1 score on the first subject.

#### 3.2.7. Offline Comparison

We compared data stream algorithms with an offline *k*-NN. The values of *k* were selected using a grid search.

### 3.3. Evaluation

We computed four metrics: the F1 score, the memory footprint, the runtime, and the power usage. The F1 score and the memory footprint were computed periodically during the execution of a classifier. The power consumption and the runtime were collected at the end of each execution.

To compute the F1 score, we monitor the true positives, false positives, true negatives, and false negatives using the prequential evaluation, meaning that, with each new data point, the model is first tested and then trained. From these counts, we compute the F1 score every 50 elements. We do not apply any fading factor to attenuate errors throughout the stream. We compute the F1 score in a one-versus-all fashion for each class, averaged across all classes (macro-average, code available https://github.com/big-data-lab-team/benchmark-har-data-stream/blob/f314bf4a258e96e418e249228897d269c59cd522/src/main.cpp#L81). When a class has not been encountered yet, its F1 score is ignored. We use the F1 score rather than the accuracy because the real data sets are imbalanced.

We measure the memory footprint by reading file /proc/self/statm every 50 data points.

The runtime of a classifier is the time needed for the classifier to process the dataset. We collect the runtime reported by the *perf* command (*perf* https://perf.wiki.kernel.org/index.php/Main_Page), which includes the loading of the binary into the memory, setting up data structures, and opening the dataset file. To remove these overheads from our measurements, we use the runtime of an empty classifier that always predict a class of zero as the baseline.

We measure the average power consumed by classification algorithms with the *perf* command. The power measurement is done multiple times in a minimal environment. We use the empty classifier as a baseline.

### 3.4. Results Reproducibility

The code and datasets used in this study are available on Github at https://github.com/big-data-lab-team/benchmark-har-data-stream.git. We used release 1.0, available on Zenodo with DOI https://doi.org/10.5281/zenodo.4148947 for long-term archival. The repository contains the source code and data we used to run the experiment, including the Makefile to compile the benchmark, the Python script to organize the execution and plot the results, and the datasets. This experiment requires the following software to run properly.

- Git to download the repository and its submodules.
- gcc or any C++ compiler in combination with make to compile the benchmark binaries.
- log4cpp as a dependency of StreamDM-C++.
- Python, seaborn, matplotlib, and pandas, to run the experiment and plot the results.

The plotting script (makefile.py) extracts data from three CSV files: models.csv, output_runs, and output. models.csv lists the combination of classifiers, datasets, and parameters that were run. This combination is called a model. The output_runs file stores information about the model’s repetition such as the runtime or the energy. Finally, the output file contains the accuracy, the F1 score, and the memory footprint every fifty elements. Each line is identified with three IDs: the model ID, the repetition count, and the data point count in the dataset.

In the README file, we provided the commands to download, compile, run the experiment, and plot the results.

During the benchmark execution, the datasets and output files were stored in memory through a memfs filesystem mounted on /tmp, to reduce the impact of I/O time. We averaged metrics across repetitions (same classifier, same parameters, and same dataset). The experiment was done with a single core of a cluster node with two Intel(R) Xeon(R) Gold 6130 CPUs and a main memory of 250 G. The node was running a CentOS Linux 8 with Linux kernel 4.18.

## 4. Results

This section presents our benchmark results and the corresponding hyperparameter tuning experiments.

### 4.1. Overall Classification Performance

**Figure 1:**
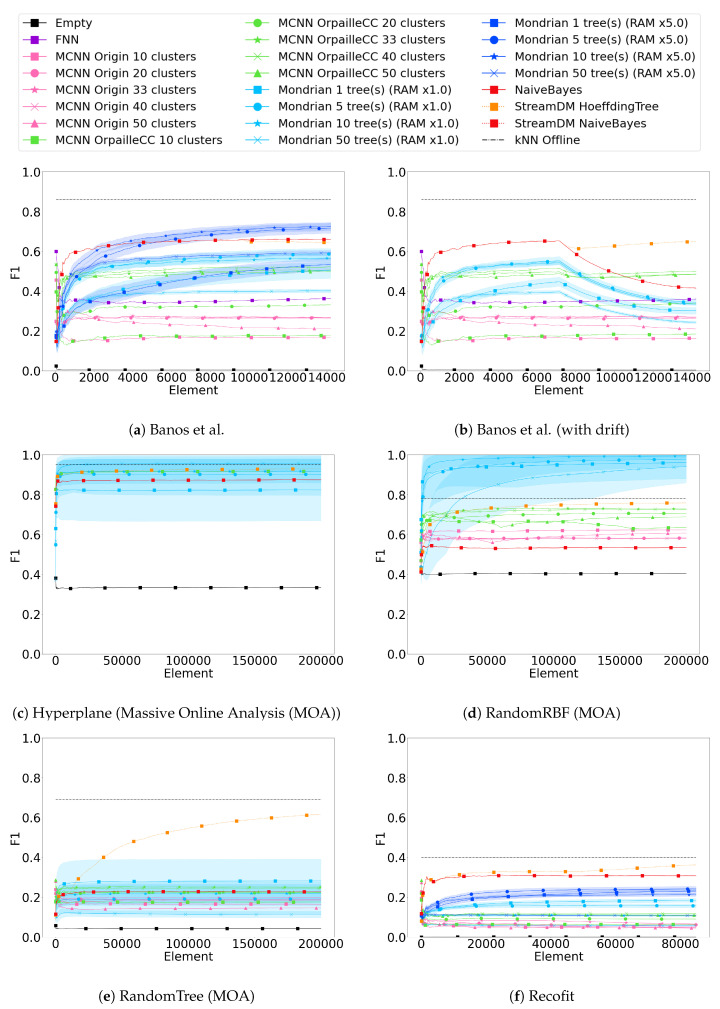
F1 scores for the six datasets (average over 20 repetitions). The horizontal dashed black line indicates the offline k-nearest neighbor (*k*-NN) F1 score (the value of k was obtained by grid search in [2, 20]). The blue shades represent a ±σ interval of the Mondrian forest classifier across repetitions.

Figure 1 compares the F1 scores obtained by all classifiers on the six datasets. The graphs also show the standard deviation of the Mondrian forest classifier observed across all repetitions (the other classifiers do not involve any randomness).

Table 3 also shows the last F1 scores obtained on each dataset.

F1 scores vary greatly across the datasets. While the highest observed F1 score is above 0.95 on the Hyperplane and RandomRBF datasets, it barely reaches 0.65 for the Banos et al. dataset, and it remains under 0.40 on the Recofit and RandomTree datasets. This trend is consistent for all classifiers.

The offline *k*-NN classifier used as the baseline achieves better F1 scores than all other classifiers, except for Mondrian forest on the Hyperplane and the RandomRBF datasets. On the Banos et al. dataset, the difference of 0.23 with the best stream classifier remains very substantial, which quantifies the remaining performance gap between data stream and offline classifiers. On the Recofit dataset, the difference between stream and offline classifiers is lower, but the offline performance remains very low.

It should be noted that the F1 scores observed for the offline *k*-NN classifier on the real datasets are substantially lower than the values reported in the literature. On the Banos et al. dataset, the original study in [28] reports an F1 score of 0.96, the work in [30] achieves 0.92, but our benchmark only achieves 0.86. Similarly, on the Recofit dataset, the original study reports an accuracy of 0.99 and the work in [30] reaches 0.65 while our benchmark only achieves 0.40. This is most likely due to our use of data coming from a single sensor, consistent with our motivating use case, while the other works used multiple ones (nine in the case of Banos et al.).

The Hoeffding Tree appears to be the most robust to concept drifts (Banos et al. with drift), while the Mondrian forest and Naïve Bayes classifiers are the most impacted. MCNN classifiers are marginally impacted. The low resilience of Mondrian forest to concept drifts can be attributed to two factors. First, existing nodes in trees of a Mondrian forest cannot be updated. Second, when the memory limit is reached, Mondrian trees cannot grow or reshape their structure anymore.

### 4.2. Hoeffding Tree and Naïve Bayes

The Naïve Bayes and the Hoeffding Tree classifiers stand out on the two real datasets (Banos et al. and Recofit) even though the F1 scores observed remain low (0.6 and 0.35) compared to offline *k*-NN (0.86 and 0.40). Additionally, the Hoeffding Tree performs outstandingly on the RandomTree dataset and Banos et al. dataset with drift. Such good performances were expected on the RandomTree dataset because it was generated based on a tree structure.

Except for the Banos et al. dataset, the Hoeffding Tree performs better than Naïve Bayes. For all datasets, the performance of both classifiers is comparable at the beginning of the stream, because the Hoeffding Tree uses Naïve Bayes in its leaves. However, F1 scores diverge throughout the stream, most likely because of the Hoeffding Tree’s ability to reshape its tree structure. This occurs after a sufficient amount of elements, and the difference is more noticeable after a concept drift.

Finally, we note that the StreamDM-C++ and OrpailleCC implementations of Naïve Bayes are indistinguishable from each other, which confirms the correctness of our implementation in OrpailleCC.

### 4.3. Mondrian Forest

On two synthetic datasets, Hyperplane and RandomRBF, the Mondrian forest (RAM × 1.0) with 10 trees achieves the best performance (F1 > 0.95), above offline *k*-NN. Additionally, the Mondrian forest with five or 10 trees ranks third on the two real datasets.

Surprisingly, a Mondrian forest with 50 trees performs worse than five or 10 trees on most datasets. The only exception is the Hyperplane dataset where a Mondrian forest with 50 trees performs than five or 10 trees. This is due to the fact that our Mondrian forest implementation is memory-bounded, which is useful on connected objects but limits tree growth when the allocated memory is full. Because 50 trees fill the memory faster than five or 10 trees, the learning stops earlier, before the trees can learn enough from the data. It can also be noted that the variance of the F1 score decreases with the number of trees, as expected.

The dependency of the Mondrian forest on memory allocation is shown in Banos et al. and Recofit datasets, where an additional configuration with five times more memory than the initial configuration was run (total of 3 MB). The memory increase induces an F1 score difference greater than 0.1, except when only one tree is used, in which case the improvement caused by the memory is less than 0.05. Naturally, the selected memory bound may not be achievable on a connected object. Overall, Mondrian forest seems to be a viable alternative to Naïve Bayes or the Hoeffding Tree for HAR.

### 4.4. MCNN

The MCNN OrpailleCC stands out on the Banos et al. (with drift) dataset where it ranks second thanks to its adaptation to the concept drift. On other datasets, MCNN OrpailleCC ranks below the Mondrian forest and the Hoeffding Tree, but above MCNN Origin. This difference between the two MCNN implementations is presumably due to the fact that MCNN Origin removes clusters with low participation too early. On the real datasets (Banos et al. and Recofit), we notice that the MCNN OrpailleCC appears to be learning faster than the Mondrian forest, although the Mondrian forest catches up after a few thousand elements. Finally, we note that MCNN remains substantially lower than the offline *k*-NN.

### 4.5. Feedforward Neural Network

Figure 1a shows that the Feedforward Neural Network has a low F1 score (0.36) compared to other classifiers (above 0.5), which contradicts the results reported in [18] where the Feedforward Neural Network achieves more than 95% accuracy in a context of offline training. The main difference between [18] and our study lie in the definition of the training set. In [18], the training set includes examples from every subject, while we only use a single one, to ensure an objective comparison with the other stream classifiers that do not require offline training (except for hyperparameter tuning, done on the first subject of the Banos et al. dataset). When we use a random sample of 10% of the datapoints across all subjects for offline training, we reach an F1 score of 0.68, which is higher than the performance of the Naïve Bayes classifier.

### 4.6. Power

**Figure 2:**
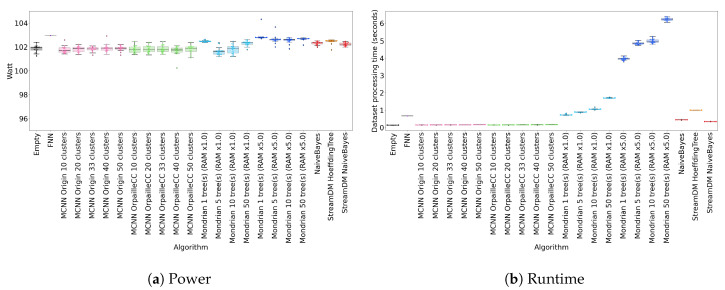
Power usage and runtime (20 repetitions) for the Banos et al. dataset. The results are similar across datasets.

Figure 2a shows the power usage of each classifier on four datasets (the results are similar for the other two datasets). All classifiers exhibit comparable power consumption, close to 102 W.

This observation is explained by two factors. First, the benchmarking platform was working at minimal power. To ensure no disturbance by a background process, we run the classifiers on an isolated cluster node with eight cores. Therefore, the power difference on one core is not noticeable.

Another reason is the dataset sizes. Indeed, the slowest run is about 10 s with 50 Mondrian trees on the Recofit dataset. Such short executions do not leave time for the CPU to switch P-states because it barely warms the core. Further experiments would be required to check how our power consumption observations generalize to connected objects.

### 4.7. Runtime

Figure 2b shows the classifier runtimes for the two real datasets. The Mondrian forest is the slowest classifier, particularly for 50 trees, which reach 2 s on the Banos et al. dataset. This represents roughly 0.35 ms/element with a slower CPU. The second slowest classifier is the Hoeffding Tree, with a runtime comparable to the Mondrian forest with 10 trees. The Hoeffding Tree is followed by the two Naïve Bayes implementations, which is not surprising since Naïve Bayes classifiers are used in the leaves of the Hoeffding Tree. The MCNN classifiers are the fastest ones, with a runtime very close to the empty classifier. Note that allocating more memory to the Mondrian forest substantially increases runtime.

We observe that the runtime of StreamDM-C++’s Naïve Bayes is comparable to OrpailleCC’s. This suggests that the performance of the two libraries is similar, which justifies our comparison of Hoeffding Tree and Mondrian forest.

### 4.8. Memory

**Figure 3:**
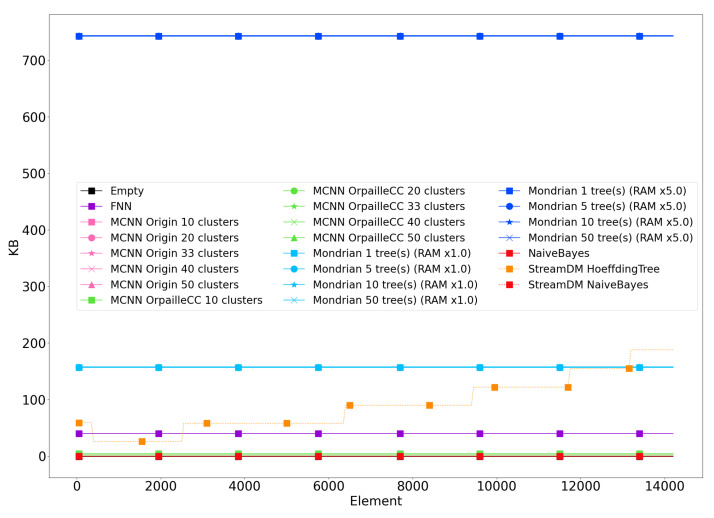
Memory footprint of classifiers with the empty classifier as a baseline, measured on the Banos et al. dataset. The memory footprint of the empty classifier is 3.44 MB. The baselines are the two Naïve Bayes from OrpailleCC and StreamDM-C++. Their respective memory footprints are 3.44 MB and 4.74 MB.

Figure 3 shows the evolution of the memory footprint for the Banos et al. dataset. The results are similar for the other datasets and are not reported for brevity. Since the memory footprint of the Naïve Bayes classifier was almost indistinguishable from the empty classifier, we used the two Naïve Bayes as a baseline for the two libraries. This enables us to remove the 1.2 MB overhead induced by StreamDM-C++. The StreamDM-C++ memory footprint matches the result in [5], where the Hoeffding Tree shows a memory footprint of 4.8 MB.

We observe that the memory footprints of the Mondrian forest and the Hoeffding Tree are substantially higher than for the other classifiers, which makes their deployment on connected objects challenging. Overall, memory footprints are similar across datasets, due to the fact that most algorithms follow a bounded memory policy or have a constant space complexity. The only exception is the Hoeffding Tree that constantly selects new splits depending on new data points. The Mondrian forest has the same behavior but the OrpailleCC implementation is memory bounded, which makes its memory footprint constant.

### 4.9. Hyperparameter Tuning

**Figure 4:**
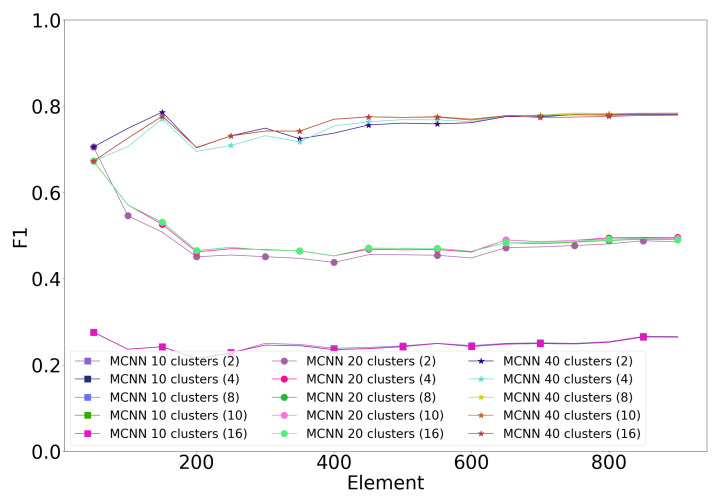
Error threshold tuning of MCNN with the first subject of Banos et al. dataset. Error threshold in parentheses.

Figure 4 shows the impact of the error threshold on the MCNN classifiers with different cluster counts. The error threshold of MCNN has little impact on classification performance. For 20 and 40 clusters, the best-performing threshold is either two or four, meaning that a cluster may have two or four errors before being split. For 10 clusters, all error thresholds perform equally.

**Figure 5:**
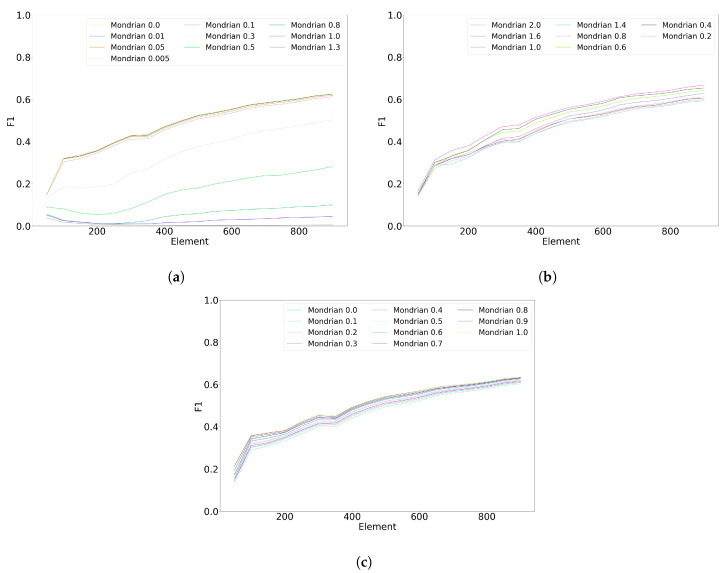
Hyperparameters tuning for Mondrian with the first subject of Banos et al. dataset. (**a**) Impact of the base count with 10 trees, a budget of 1.0, and a discount factor of 0.2. (**b**) Impact of the budget with 10 trees, a base count of 0.1, and discount factor of 0.2. (**c**) Impact of the discount factor with 10 trees, a budget of 1.0, and a base count of 0.1.

Figure 5 shows the impact of the Mondrian forest hyperparameters on the classification performance. The base count hyperparameter (Figure 5a) has a very substantial impact on classification performance; the smallest value (0.0) results in the best performance. On the contrary, the budget hyperparameter (Figure 5b) only has a moderate impact on classification; the best value is 0.2. Finally, the discount hyperparameter (Figure 5c) has a negligible impact on the performance; the best-performing value is 0.1.

## 5. Conclusions

We conclude that the Hoeffding Tree, the Mondrian forest, and the Naïve Bayes data stream classifiers have an overall superiority over the Feedforward Neural Network and the MCNN ones for HAR. However, the prediction performance remains quite low compared to an offline *k*-NN classifier, and it varies substantially between datasets. Noticeably, the Hoeffding Tree and the MCNN classifiers are more resilient to concept drift that the other ones.

Regarding memory consumption, only the MCNN and Naïve Bayes classifiers were found to have a negligible memory footprint, in the order of a few kilobytes, which is compatible with connected objects. Conversely, the memory consumed by a Mondrian forest, a Feedforward Neural Network or a Hoeffding Tree is in the order of 100 kB, which would only be available on some connected objects. In addition, the classification performance of a Mondrian forest is strongly modulated by the amount of memory allocated. With enough memory, a Mondrian forest is likely to match or exceed the performance of the Hoeffding Tree and Naïve Bayes classifiers.

The amount of energy consumed by classifiers is mostly impacted by their runtime, as all power consumptions were found to be comparable. The Hoeffding Tree and Mondrian forest are substantially slower than the other classifiers, with runtimes in the order of 0.35 ms/element, a performance not compatible with the common sampling frequencies of wearable sensors.

Our results show that even though data stream classifiers were developed to minimize memory footprint, they were not developed to work with a given memory budget. For instance, there is no guarantee that the Mondrian forest or the Hoeffding Tree would make the best split selection on a device with a small amount of memory. Future research could focus on developing algorithms that can guarantee a peak memory consumption and ensure an optimal response within a memory budget. In particular, we are planning to explore tree sampling methods based on memory and performance criteria in a Mondrian forest. In addition, deploying classification algorithms on actual connected objects might highlight other relevant research directions.

## Figures and Tables

**Table 1:** Hyperparameters used for the Mondrian forest.

Number of Trees	Base Count	Discount	Budget
1	0.0	1.0	1.0
5	0.0	1.0	0.4
10	0.0	1.0	0.4
50	0.0	1.0	0.2

**Table 2:** Hyperparameters used for the Micro-Cluster Nearest Neighbor (MCNN).

Number of Clusters	Error Threshold	Participation Threshold
10	2	10
20	10	10
33	16	10
40	8	10
50	2	10

**Table 3:** Average F1 scores obtained on the last data point of the stream.

	Hyperplane	RandomRBF	RandomTree	Recofit	Banos et al.	Banos et al. (Drift)
Empty	0.333	0.404	0.041	0.000	0.004	0.004
MCNN Origin 10 clusters	0.918	0.624	0.164	0.050	0.169	0.163
MCNN Origin 20 clusters	0.918	0.583	0.188	0.061	0.265	0.262
MCNN Origin 33 clusters	0.918	0.584	0.196	0.065	0.268	0.270
MCNN Origin 40 clusters	0.918	0.581	0.183	0.047	0.266	0.268
MCNN Origin 50 clusters	0.918	0.607	0.146	0.045	0.212	0.210
MCNN OrpailleCC 10 clusters	0.918	0.637	0.176	0.063	0.178	0.185
MCNN OrpailleCC 20 clusters	0.918	0.707	0.222	0.090	0.332	0.339
MCNN OrpailleCC 33 clusters	0.918	0.729	0.250	0.109	0.507	0.487
MCNN OrpailleCC 40 clusters	0.918	0.725	0.244	0.118	0.522	0.501
MCNN OrpailleCC 50 clusters	0.918	0.689	0.226	0.109	0.500	0.485
Mondrian 1 tree(s) (RAM ×1.0)	0.825	0.961	0.282	0.183	0.506	0.302
Mondrian 5 tree(s) (RAM ×1.0)	0.903	0.978	0.190	0.157	0.588	0.345
Mondrian 10 tree(s) (RAM ×1.0)	0.919	0.994	0.218	0.107	0.566	0.336
Mondrian 50 tree(s) (RAM ×1.0)	0.957	0.942	0.112	0.056	0.402	0.244
NaiveBayes	0.875	0.534	0.227	0.307	0.661	0.416
StreamDM HoeffdingTree	0.931	0.759	0.617	0.362	0.656	0.650
StreamDM NaiveBayes	0.875	0.534	0.227	0.311	0.661	0.416
Mondrian 1 tree(s) (RAM ×5.0)				0.230	0.536	
Mondrian 5 tree(s) (RAM ×5.0)				0.241	0.717	
Mondrian 10 tree(s) (RAM ×5.0)				0.212	0.726	
Mondrian 50 tree(s) (RAM ×5.0)				0.107	0.593	
Feedforward Neural Network					0.365	0.360
